# Attitudes and Behaviors of the Public Toward over the Counter (OTC) Medications in the Western Region of Saudi Arabia: A Cross-Sectional Study

**DOI:** 10.3390/healthcare13050472

**Published:** 2025-02-21

**Authors:** Mohammed S. Alharthi, Fahad T. Alsulami, Ahmed Ibrahim Fathelrahman, Majed A. Alqarni, Nasser M. Alorfi, Mohammed S. Alzahrani

**Affiliations:** 1 Department of Clinical Pharmacy, College of Pharmacy, Taif University, Taif 21944, Saudi Arabia; f.alsulami@tu.edu.sa (F.T.A.); aihassan@tu.edu.sa (A.I.F.); m.alqarni@tu.edu.sa (M.A.A.); m.s.alzahrani@tu.edu.sa (M.S.A.); 2 Pharmacology and Toxicology Department, College of Pharmacy, Umm Al-Qura University, Makkah 24382, Saudi Arabia; nmorfi@uqu.edu.sa

**Keywords:** over the counter medications, self-care, attitudes, behaviors, public, Western region, Saudi Arabia

## Abstract

**Background:** Over the counter (OTC) medications are readily accessible to the public and can be obtained without a prescription for self-care. Ensuring their safe and effective use is essential to safeguarding the well-being of the region’s diverse population. This research explores public attitudes and behaviors toward OTC medications in the Western Region of Saudi Arabia in order to inform and advance public health initiatives. **Method:** This cross-sectional study was conducted in the Western Region of Saudi Arabia. A validated questionnaire was used to survey 200 respondents. The collected data were analyzed using frequencies and percentages. The demographic–OTC medication purchase relationship was examined using the chi-square test. **Results:** Among the 200 participants, 79.5% were aware of potential drug interactions, and 74% supported stricter regulations on OTC sales. However, only 29.5% believed OTC medications are generally safe without a doctor’s prescription. Notably, 15.5% reported exceeding recommended dosages, and 66% admitted sharing medications. Participants commonly relied on healthcare professionals for advice (64.6%). Despite moderate knowledge, many exhibited risky behaviors, highlighting gaps in consumer awareness and safety practices. **Conclusions:** It is critical to determine the degree of public knowledge in this field in order to identify gaps in consumer awareness of OTC medications and specific areas that may demand targeted educational programs in Saudi Arabia. Information on potential side effects and circumstances that exclude the use of over the counter medications must also be examined. Attitudes towards symptom management and a tendency for self-medication may influence the use of OTC as an alternative to seeking medical advice.

## 1. Introduction

Over the counter (OTC) medications, available without a prescription, are integral to modern healthcare, enabling individuals to independently manage minor ailments, such as headaches, colds, coughs, and allergies. Their accessibility and affordability make them a cornerstone of self-care, offering immediate symptom relief while reducing the burden on healthcare systems. OTC medications empower patients to manage their health actively, fostering greater health literacy and self-reliance. However, their widespread availability also raises concerns about misuse, drug interactions, and the need for appropriate guidance. Understanding the usage patterns, attitudes, and behaviors associated with OTC medications is essential to inform regulatory policies and public health initiatives. This ensures their safe and effective use while maximizing their contribution to public health [1].

A cross-sectional study exploring attitudes and behaviors toward OTC medications found that 24.6% of respondents reported regular OTC use [2]. While patients expressed positive attitudes toward doctors inquiring about prior OTC use and recommending OTC options, they were less receptive to pharmacists making generic or therapeutic substitutions. These findings highlight the importance of understanding public attitudes and behaviors toward OTC medications in order to inform healthcare practices. In a study in Poland, one-third of participants purchased OTC medications less than once every three months, and more than half purchased OTC medications for colds [3]. Most respondents believed OTC medications should only be available in pharmacies. At least 40% of senior citizens used an OTC medicine, with many relying on physicians and pharmacists for guidance. Additionally, over 75% of respondents knew the correct way to dispose of unused OTC medications.

A 2021 systematic review examined misuse, abuse, and dependence on OTC medications, identifying medications involved, contributory variables, harm caused, and risk-mitigation strategies [4]. The review included 53 of 2355 peer-reviewed papers and reported pooled incidences of 16.2% for misuse, 2.0% for abuse, and 7.2% for dependence. The most problematic OTC medications were analgesics (with or without codeine), sedative antihistamines, and dextromethorphan cough combinations. Misuse led to physical, psychological, social, and financial suffering, with extreme cases resulting in hospitalization or death. In Japan, researchers found that national OTC brands were valued more highly than private brands, potentially influenced by advertising [5]. Participants of lower socioeconomic status prioritized factors other than effectiveness or components when choosing OTC medications. Many Japanese participants lacked awareness of potential misuse and adverse effects. Despite these considerations surrounding OTC medications, the factors affecting consumer decisions remain complex and challenging to isolate [6]. While a wealth of research provides insights into OTC medication usage [7,8,9,10], significant gaps remain in understanding the relationships between consumers and this accessible class of medication.

In Saudi Arabia, studies have highlighted various aspects of OTC medication use. OTC analgesics are the most commonly used, with diclofenac being the most frequently utilized [11]. The misuse and abuse of medications, such as sedative antihistamines, dextromethorphan-containing cough products, and codeine-based analgesics, are prevalent, driven by self-management of medical conditions and, in some cases, recreational use or sleep induction [12]. Community pharmacists noted frequent misuse and advocated rescheduling of high-risk medications to prescription-only status [12]. Among parents, a study in Jeddah reported that 58.8% purchased OTC medications from community pharmacies for their children, with family physicians being the most common source of information (37.3%) [13].

In Saudi Arabia, OTC medications are widely available in community pharmacies and include various categories such as analgesics (e.g., paracetamol, ibuprofen, diclofenac), antipyretics, antihistamines (e.g., loratadine, cetirizine), cough and cold preparations (e.g., dextromethorphan, guaifenesin), antacids and gastrointestinal medications (e.g., omeprazole, ranitidine, loperamide), and topical agents (e.g., hydrocortisone creams, antifungal treatments). These medications manage common health conditions, such as fever, allergies, minor pain, and digestive disturbances. However, their widespread availability raises concerns about potential misuse, drug interactions, and the need for public education on appropriate use [14].

The current study conducts a detailed cross-sectional examination of factors influencing attitudes and behaviors toward OTC medications in the Western Region of Saudi Arabia, chosen for its unique demographic and geographic significance. The region includes major urban centres, such as Jeddah, Makkah, and Taif, characterized by a diverse population and a mix of urban and rural communities. As a hub for millions of residents and visitors, driven by its religious, economic, and cultural importance, the Western Region provides an ideal setting for exploring public health behaviors. By assessing consumer knowledge, attitudes, and behaviors, this study identifies critical gaps requiring targeted educational initiatives. Additionally, it evaluates awareness of potential adverse effects, contraindications, and attitudes toward symptom management and self-medication. These findings aim to inform healthcare practices, regulatory policies, and public health strategies, contributing to improved health outcomes in the community and offering insights applicable to other regions with similar dynamics.

## 2. Methods

### 2.1. Study Setting

A cross-sectional study was conducted by distributing an online questionnaire to the public in the Western Region of Saudi Arabia. Convenient sampling was used to conduct the study and allow for more effective survey distribution among the Saudi Arabian public in the Western region. All members of the public who were eighteen years old and over were included. Although the Western Region of Saudi Arabia includes both urban and rural populations, the survey did not specifically ask about participants’ place of residence. The study aimed to capture general public perspectives rather than focus on urban–rural differences. Future research could further explore how urban or rural backgrounds influence OTC medication use and attitudes.

### 2.2. Sample Size

The sample size was estimated using the formula n = z2*p* (1 − *p*)/10^2^, assuming a 95% confidence interval (z-score = 1.96), a 5% margin of error, and an estimated population proportion (*p*) of 0.5. This calculation initially suggested a sample size of 384; however, a final sample size of 200 was chosen due to practical constraints.

### 2.3. Validity and Reliability

The questionnaire used in this study was developed to align with the study’s aim and objectives, drawing from a comprehensive review of relevant literature [15,16,17]. Two academic experts evaluated the questionnaire to ensure its accuracy, relevance, and appropriateness. To assess its validity, the questionnaire was piloted with a sample of 15 individuals representing a range of demographic characteristics, including age and education levels. Feedback from the pilot study was incorporated into the final version of the questionnaire, though the pilot results were excluded from the main analysis. To ensure broad accessibility and high response rates, the questionnaire was distributed through social media platforms, including X v. 10.81.2 and WhatsApp v.25.2.79. Participants provided informed consent before beginning the survey.

### 2.4. Measurement of Constructs

The questionnaire comprised three key constructs: knowledge, attitudes, and behaviour related to OTC medications. While knowledge and attitudes were assessed through perception-based questions, behaviour-related items were added to capture actual practices, such as medication sharing and expiration date tracking. The knowledge construct included five items that assessed participants’ awareness of critical aspects of OTC medication use, such as drug interactions, safety considerations, cost-effectiveness, and regulatory requirements. The attitudes construct included questions to capture participants’ perspectives on various aspects of OTC medication use. Participants responded using a three-point scale with options such as ‘Yes’, ‘Unsure’, and ‘No’. Responses were scored to reflect levels of knowledge, with correct answers assigned higher scores. The overall knowledge score was calculated as the mean of the individual item scores, with scores categorized as adequate knowledge (2.34–3.00), moderately adequate knowledge (1.67–2.33), or inadequate knowledge (1.00–1.66).

Attitudes were assessed using a combination of three-point and four-point Likert scales, depending on the nature of the question. While most items were rated on a three-point scale (e.g., confident, somewhat confident, not confident), some questions used a four-point scale to capture a broader range of responses (e.g., strongly agree, agree, disagree, strongly disagree). These items explored participants’ perspectives on various aspects of OTC medication use, including confidence in selecting medications, consulting healthcare professionals, and concerns about long-term safety. Individual responses were averaged to produce an overall attitude score, which was interpreted as strong (2.34–3.00), average (1.67–2.33), or weak (1.00–1.66).

In contrast, behaviour-related items, such as keeping track of expiration dates and sharing OTC medications, were categorized separately to distinguish practical medication use from perceptions and beliefs. Knowledge and attitudes were analysed using three-point categorical scales, while behaviors were assessed using binary (Yes/No) and multiple-choice items. This classification ensures a more straightforward interpretation of participants’ self-reported medication practices versus their perceptions and beliefs regarding OTC use. Some survey questions were designed to assess participants’ perceptions and opinions rather than objective facts, for example, questions regarding the safety of OTC medications and the importance of having a wide variety of options, in order to capture public perspectives rather than determine absolute correctness.

### 2.5. Ethical Requirements

Taif University’s Committee granted ethical approval for this study under application 45-162. The study adhered to key ethical principles, including ensuring confidentiality and obtaining informed consent. Before consent, participants were thoroughly briefed on the study’s objectives, procedures, potential risks, and benefits. Their participation was entirely voluntary and free from any form of coercion. Furthermore, the research team took measures to safeguard participants’ privacy and protect their identities and sensitive data.

### 2.6. Data Analysis

The data were collected through an online questionnaire using Microsoft Forms and entered into Statistical Package for the Social Sciences (SPSS) version 26 to analyze the basic features of the collected data through frequencies and percentages. The chi-square test was used to find the association between demographics and OTC medication purchasing habits. *t*-test was used to compare means between different groups, such as comparing attitudes towards OTC medications between different demographic groups. Cross-tabulation with chi-square test were used to determine the factors influencing the choice of OTC medications. A *p* value of <0.05 was considered statistically significant.

## 3. Results

A total of 200 participants from the Western Region of Saudi Arabia were included in the study. Table 1 shows that the sample was predominantly male (n = 140, 70.0%) compared to female (n = 60, 30.0%). Regarding age distribution, the highest proportion of male participants belonged to the 26–35 age group (n = 42, 30.0%), followed by those aged 36–45 years (n = 38, 27.1%). Among females, the most common age group was also 36–45 years (n = 19, 31.7%), followed by 26–35 years (n = 16, 26.7%). Regarding educational background, more than half of the participants (54.5%) had a bachelor’s degree. Among males, 53.6% held a bachelor’s degree, while 56.7% of females had attained the same level of education. Participants with higher education degrees (master’s or doctorate) comprised 18.5% of the sample, with 19.3% of males and 16.7% of females falling into this category. These findings provide a clearer picture of the demographic composition of the participants and help contextualize the study’s analysis of OTC medication use, behaviors and attitudes. Regarding gender distribution, males constituted a more significant proportion of the sample (70%), which may reflect differences in survey accessibility or willingness to participate. Efforts were made to ensure broad representation through online distribution and outreach; however, cultural and social factors may have influenced participation rates.

### 3.1. Knowledge of OTC Medications

Table 2 shows that the majority of participants were aware of potential drug interactions (79.5%) and supported stricter regulations on OTC medications (74%). However, only 29.5% believed OTC medications are generally safe for use without a doctor’s prescription, while 42.5% disagreed. Regarding cost-effectiveness, 48.5% agreed that OTC medications are a cost-effective alternative, whereas 51.5% disagreed. Additionally, 43% did not believe that OTC advertisements accurately represent the benefits and risks, and 37% were unsure.

### 3.2. Knowledge Mean Score

As shown in Table 3, the study utilized a three-point scale to assess the level of knowledge regarding OTC medications, with the scale interpreted as follows: 3 indicating adequate knowledge (2.34–3.00), 2 representing moderately adequate knowledge (1.67–2.33), and 1 signifying inadequate knowledge (1.00–1.66). The mean score obtained was 2.28, reflecting a moderately adequate knowledge level, corresponding to a 76% understanding of OTC medications. Participants demonstrated a moderate to adequate knowledge of OTC medications, with an overall mean score of 2.28. The highest knowledge was related to drug interactions with prescription drugs (Mean = 2.60), while the lowest was observed for the importance of variety in OTC options (Mean = 1.84). This variation highlights differing levels of awareness across various aspects of OTC medication use.

### 3.3. Attitudes Towards OTC Medications

Table 4 demonstrates that most participants reported confidence in selecting appropriate OTC medications (74%), with healthcare professionals identified as the most frequently utilized source of information (64.6%). Some attitude-related questions were assessed using a three-point scale, while others used a four-point scale to capture participant perspectives better. Additionally, a question regarding the importance of having a wide variety of OTC medication options, previously categorized under knowledge, has been reassigned to attitudes, as it reflects participant perceptions rather than factual knowledge. Despite expressing considerable concern regarding the potential long-term adverse effects of unsupervised OTC use (61.5%), a notable proportion (15.5%) indicated exceeding the recommended dosages. Pharmacies were the preferred point of purchase (88.5%), and generic products were chosen over brand-name options by more than half of the respondents (54%). Additionally, a significant percentage (66%) acknowledged engaging in the practice of sharing OTC medications. Interestingly, only a minority (33%) expressed a willingness to pay a premium for products labelled as “organic” or “natural”, indicating limited interest in such attributes.

### 3.4. Attitude Mean Score

As shown in Table 5, the three-point scale used in the study categorized attitudes as follows: positive (2.34–3.00), neutral (1.67–2.33), and negative (1.00–1.66). The overall mean score of 2.29 indicated a 76% level of attitude regarding OTC medications, suggesting a generally neutral stance. Participants showed the most positive attitudes toward their preferred purchase locations (M = 2.82) and confidence in choosing OTC products (M = 2.71). Positive attitudes were also observed in consulting healthcare professionals (M = 2.64) and tracking expiration dates (M = 2.63). Neutral attitudes were reflected in use of information sources, willingness to pay more for “organic” labels, and choosing between generic or brand-name options. The lowest scores were related to exceeding dosages (M = 1.76) and concerns about long-term effects (M = 1.75). Sharing OTC medications (M = 1.67) ranked the lowest, indicating a neutral to negative attitude.

### 3.5. Behavior-Related Items

In addition to knowledge and attitudes, we assessed behaviour-related items to distinguish practical medication practices from perceptions. Table 6 shows that a significant proportion of participants (66%) reported sharing OTC medications with others, posing risks of improper dosing and adverse drug reactions. Additionally, 81% of participants stated that they track the expiration dates of OTC medications, demonstrating a generally responsible approach to medication storage.

### 3.6. Behavior Mean Score

In addition to assessing knowledge and attitudes toward OTC medications, this study also examined specific behaviours related to medication use. These behaviours include practices such as sharing medications and tracking expiration dates. Table 7 shows that the mean behaviour score was 2.22, indicating a generally moderate level of responsible behaviour among participants. While the majority reported tracking medication expiration dates, a significant proportion still engaged in the risky practice of sharing medications. This highlights the need for targeted public health interventions to enhance safe medication practices.

### 3.7. Association of Socio-Demographic Factors with Participants’ Knowledge and Attitudes Towards OTC Medications

Table 8 indicates that there was no statistically significant association between participants’ age, gender, or education level and their overall level of knowledge regarding OTC medications, as none of the *p*-values fell below the 0.05 threshold. However, a trend was observed, suggesting that participants with a bachelor’s degree or higher tended to have a higher proportion of adequate knowledge compared to those with lower educational backgrounds. Similarly, while younger participants aged 26 to 35 years showed relatively higher knowledge levels compared to older age groups, these differences did not reach statistical significance. Additionally, gender differences in knowledge levels appeared minimal, with both male and female participants showing similar distributions across the knowledge categories.

Table 9 indicates no statistically significant association between participants’ age, gender, or education level and their overall attitude towards OTC medications, as all *p*-values exceeded the 0.05 threshold. However, a trend was observed in which participants aged 36 to 45 demonstrated a slightly higher proportion of positive attitudes than other age groups. Additionally, gender differences were minimal, with similar distributions of positive and neutral attitudes between males and females. Participants with higher education degrees tended to exhibit more positive attitudes than those with a high school or lower education, though these differences were not statistically significant.

## 4. Discussion

This study provides an in-depth exploration of the attitudes and behaviors of the Western Region of Saudi Arabian public regarding over the counter (OTC) medications. The key findings revealed that the majority of participants possessed moderately adequate knowledge of OTC medications, particularly regarding drug interactions with prescription medications. However, there were notable gaps in understanding other aspects of OTC use, such as the importance of having various OTC medication options. Regarding attitudes, most participants expressed confidence in selecting the appropriate OTC medication for self-care, with healthcare professionals serving as their primary source of information. Nevertheless, participants demonstrated mixed attitudes towards the long-term safety of OTC medications and showed a neutral stance regarding exceeding recommended dosages and sharing medications. Furthermore, demographic factors, including age, gender, and education, did not have a statistically significant association with participants’ overall knowledge or attitudes towards OTC medications.

The findings of this study align with existing literature on public awareness and use of OTC medications [18,19,20], but they also introduce new insights that are specific to the Saudi Arabian public. Similar to studies conducted in other countries, such as Poland and Sweden [21,22], the Saudi Arabian public demonstrated a high awareness of the potential for drug interactions between OTC and prescription medications. This highlights a general understanding of the risks involved in unsupervised medication use, which is consistent with global trends in healthcare consumerism that emphasize safety and responsibility in self-medication [23]. This awareness is crucial given the widespread availability of OTC drugs and the increasing reliance on self-care practices in many parts of the world. A 2021 systematic review similarly emphasized the risks associated with misuse and abuse of certain OTC medications, particularly Antihistamines, Cough Medicines, and Decongestants, suggesting that consumers must be vigilant about their use of these medications [24]. Recent studies indicate that consumers often prioritize factors such as efficacy, cost, and availability over the diversity of over the counter (OTC) medication options. For instance, a 2024 study found that 93% of U.S. adults prefer to treat their minor ailments with OTC medicines before seeking professional care, highlighting a preference for convenience and immediate access. Additionally, 96% of adults believe OTC medicines make it easy to care for minor medical ailments, underscoring the importance of accessibility and effectiveness in consumer decision-making [19]. The public in the Western Region of Saudi Arabia, like their counterparts in other regions, appears to base its choices on immediate needs and practical considerations, rather than on an informed understanding of the full range of available OTC options. This knowledge gap may reflect the need for more accessible public education campaigns that inform consumers not only about the dangers of misuse but also about the importance of understanding product variety and options. Participants’ confidence in choosing OTC medications is likely attributable to their frequent reliance on healthcare professionals for information. Healthcare professionals are identified as the most trusted and frequently used source of information for over the counter (OTC) products. This trust in healthcare professionals is consistent with findings from international studies. For instance, a survey conducted in Japan revealed that consumers prefer obtaining medication guidance in pharmacies or stores over other approaches, indicating a reliance on healthcare providers for advice on OTC medication use [25]. This trend suggests that the Saudi public values professional medical advice, even in the context of self-care and over-the-counter remedies. This is encouraging from a public health perspective, as it can mitigate the risks associated with unsupervised use. Participants’ mixed attitudes toward the long-term safety of OTC medications align with global findings, as seen in a study in Al-Ahsa, Saudi Arabia, which highlighted concerns about misuse despite the perceived safety of these drugs, and in research from Canada, where consumers viewed OTC medications as effective but had low awareness of long-term risks, emphasizing the need for public education on responsible use and the importance of consulting healthcare professionals [26,27]. A potential explanation for this could be the relatively easy access to OTC medications in pharmacies without stringent regulations or limitations. While most respondents are aware of the possibility of adverse effects or drug interactions, this awareness does not necessarily translate into cautious behavior, as indicated by the notable percentage of participants who exceed recommended dosages and share medications with others. This pattern of behavior is not unique to Saudi Arabia and has been observed in other parts of the world, where public education efforts on the risks of OTC misuse are still lacking [28]. The prevalence of medication-sharing among the Saudi Arabian public is a particularly noteworthy finding. While sharing medications is often viewed as an act of care or convenience, it presents serious public health risks, including improper dosing and increased chances of adverse drug reactions. Cultural factors may influence this behavior, as social norms in Saudi Arabia may promote medication-sharing as a form of assistance within families and communities. This finding aligns with other studies in regions where familial and social bonds are strong and the informal exchange of medical supplies is more common. For instance, a study in Saudi Arabia found that various medications were borrowed and lent mainly between immediate family members, with various reasons identified for this behaviour [29]. Addressing this behaviour will require culturally sensitive public health campaigns highlighting the risks of sharing medications and providing alternative means of support and care within communities. The study’s inability to detect statistically significant associations between demographic factors (such as age, gender, or education level) and participants’ knowledge or attitudes towards OTC medications may be due to the relative homogeneity of healthcare literacy across the population. While younger participants and those with higher education levels exhibited slightly higher knowledge levels and more positive attitudes toward medication use, these differences were not substantial enough to indicate a significant disparity. This observation may reflect the widespread accessibility of healthcare information in Saudi Arabia, which transcends specific demographics. Notably, this finding contrasts with international studies that have demonstrated a strong correlation between higher educational attainment and improved health outcomes. For instance, research indicates that each additional year of education reduces the risk of death by 2%, with 18 years of education lowering the risk by 34%. Similarly, a study in the United States found that adults residing in communities with a higher percentage of college graduates experience lower mortality rates, suggesting that the benefits of education extend beyond individual attainment to community-wide health improvements [30,31]. The increasing penetration of digital health resources and the widespread influence of social media in Saudi Arabia may have helped bridge the knowledge gap between different demographic groups. The study findings emphasize the need for targeted educational campaigns that focus on filling the knowledge gaps regarding OTC medication use, particularly in areas where misconceptions or risky behaviors, such as sharing medications or exceeding recommended dosages, persist. In the context of growing self-care practices, it is essential to understand that, although consumers are becoming more empowered to make health-related decisions, they must be supported with accurate information to avoid potential harm.

### Strengths and Limitations of the Study

The strengths of this study lie in its comprehensive overview of the attitudes and behaviors of the Saudi Arabian public regarding OTC medications. The use of an online survey allowed for a broad and geographically diverse distribution, ensuring that different segments of the population were represented. Additionally, the questionnaire was carefully developed and validated, ensuring that it accurately captured the variables of interest. However, the study has some limitations. Convenient sampling is less representative of the population compared to probability sampling techniques. However, taking a probability sample was not possible due to absence of sampling frame. Self-reporting is less accurate than direct assessment in capturing actual behaviors, where participants may underreport risky behaviors, such as exceeding dosages, or over-report positive ones. We need to be cautious in interpreting findings as the smaller sample size might limit generalizability. The study might not be able to detect minor real differences between groups. Additionally, the cross-sectional design limits the ability to establish temporal relationships between variables, thus cannot prove causality between attitudes, behaviors, and demographic factors, yet can prove associations. Further prospective follow-up studies will be required to establish causality. In addition, this study does not include data on participants’ urban or rural origin, despite the Western Region’s diverse demographic composition. Future studies should consider including this variable to better understand its influence on OTC medication use. Additionally, the study had a higher proportion of male participants (70%), which may limit the generalizability of findings to the broader population. Targeted recruitment strategies could be employed in future research to ensure more balanced gender representation.

## 5. Conclusions

In conclusion, this study provides valuable insights into the attitudes and behaviors regarding OTC medications among the public in the Western Region of Saudi Arabia. While participants demonstrated moderately adequate knowledge, particularly about drug interactions, significant gaps remain in understanding product variety and the long-term risks of unsupervised OTC medication use. The findings emphasize the need for public health campaigns to address these knowledge gaps and promote safer self-care practices. Future research should explore cultural factors influencing medication-sharing behaviors and develop strategies to enhance public understanding of the potential dangers of OTC medication misuse. The insights from this study can inform healthcare professionals, policymakers, and public health initiatives, ultimately contributing to better health outcomes in Saudi Arabia.

## Figures and Tables

**Table 1:** Demographic characteristics of the study participants.

Characteristics	Total (n, %)	Male (n, %)	Female (n, %)
Age (years)			
18 to 25	41 (20.5)	30 (21.4)	11 (18.3)
26 to 35	58 (29.0)	42 (30.0)	16 (26.7)
36 to 45	57 (28.5)	38 (27.1)	19 (31.7)
46 to 55	30 (15.0)	22 (15.7)	8 (13.3)
Above 55	14 (7.0)	8 (5.7)	6 (10.0)
Highest Education Level			
High School or Less	29 (14.5)	20 (14.3)	9 (15.0)
Diploma Degree	25 (12.5)	18 (12.9)	7 (11.7)
Bachelor’s Degree	109 (54.5)	75 (53.6)	34 (56.7)
Higher Education	37 (18.5)	27 (19.3)	10 (16.7)

**Table 2:** Participants’ responses regarding knowledge of OTC medications.

Question (n = 200)	n	(%)
Do you believe OTC medications are generally safe without a doctor’s prescription?		
Yes	59	29.5
Unsure	56	28.0
No	85	42.5
Do you feel that OTC medications are a cost-effective alternative to prescription medications for common health issues?		
Strongly agree	21	10.5
Agree	76	38.0
Disagree	79	39.5
Strongly disagree	24	12.0
Do you think OTC medication advertisements accurately represent the benefits and risks of these products?		
Yes	40	20.0
Unsure	74	37.0
No	86	43.0
Not important at all	14	7.0
Do you think there should be stricter regulations on the sale of certain OTC medications?		
Yes	148	74.0
Unsure	2	1.0
No	50	25.0
Are you aware of the potential for drug interactions between OTC medications and prescription drugs?		
Yes	159	79.5
Unsure	2	1.0
No	39	19.5

**Table 3:** Participants’ level of knowledge regarding OTC medications.

Statement	Mean	SD	Rank	Level of Knowledge
Do you believe that OTC medications are generally safe for use without a doctor’s prescription?	2.13	0.841	5	Moderately adequate knowledge
Do you feel that OTC medications are a cost-effective alternative to prescription medications for common health issues?	2.41	0.674	3	Adequate knowledge
Do you think OTC medication advertisements accurately represent the benefits and risks of these products?	2.23	0.762	4	Moderately adequate knowledge
How important is it for you to have a wide variety of OTC medication options to choose from?	1.84	0.788	6	Moderately adequate knowledge
Do you think there should be stricter regulations on the sale of certain OTC medications?	2.49	0.868	2	Adequate knowledge
Are you aware of the potential for drug interactions between OTC medications and prescription drugs?	2.60	0.796	1	Adequate knowledge
Overall knowledge (Mean Score)	2.28			

**Table 4:** Participants’ responses regarding attitudes towards OTC medications.

Question (n = 200)	n	(%)
How confident do you feel in choosing the right OTC medication for your needs?		
Very confident	65	32.5
Somewhat confident	83	41.5
Not very confident	46	23.0
Not confident at all	6	3.0
What sources do you primarily rely on for information about OTC medications? (Check all that apply) (n = 200, k = 299)		
Healthcare professionals	128	64.6
Internet	71	35.9
Friends and family	68	34.3
Television advertisements	8	4.0
Other	24	12.1
How often do you exceed the recommended doses of OTC medications when self-medicating?		
Never	84	42.0
Rarely	80	40.0
Occasionally	5	2.5
Often	31	15.5
Are you concerned about the potential long-term effects of using OTC medications without medical guidance?		
Very concerned	26	13.0
Somewhat concerned	97	48.5
Not very concerned	57	28.5
Not concerned at all	20	10.0
Have you ever consulted a healthcare professional before choosing an OTC medication?		
Yes	164	82.0
Unsure	1	.5
No	35	17.5
Have you ever experienced any adverse effects from OTC medications?		
Yes	26	13.0
Unsure	44	22.0
No	130	65.0
Would you be willing to pay more for OTC medications if they were labelled as “organic” or “natural”?		
Yes	66	33.0
Unsure	2	1.0
No	132	66.0
Where do you usually purchase OTC medications?		
Pharmacies	177	88.5
Grocery stores	9	4.5
Online retailers	14	7.0
Do you usually buy generic or brand-name OTC medications?		
Generic	108	54.0
Brand-name	72	36.0
I don’t know	20	10.0
How important is it for you to have a wide variety of OTC medication options?		
Very important	48	24.0
Somewhat important	71	35.5
Not very important	67	33.5

**Table 5:** Participants’ level of attitude regarding OTC medications.

Statement	Mean	SD	Rank	Level of Attitude
How confident do you feel in choosing the right OTC medication for your needs?	2.71	0.517	2	Strong attitude
What sources do you primarily rely on for information about OTC medications?	2.17	0.76167	8	Average
How often do you exceed the recommended dosage of OTC medications when self-medicating?	1.76	0.738	9	Average
Are you concerned about the potential long-term effects of using OTC medications without medical guidance?	1.75	0.672	10	Average
Have you ever consulted a healthcare professional before choosing an OTC medication?	2.64	0.763	3	Strong attitude
Have you ever experienced any adverse effects from OTC medications?	2.52	0.716	5	Strong attitude
Would you be willing to pay more for OTC medications if they labeled as “organic” or “natural”?	2.33	0.941	6	Average
Where do you usually purchase OTC medications?	2.82	0.541	1	Strong attitude
Do you usually buy generic or brand name OTC medications?	2.18	0.934	7	Average
Overall Mean Attitude Score	2.29			

**Table 6:** Participants’ Behavior-Related Responses.

Question (n = 200)	n (%)	n (%)	n (%)
Do you keep track of the expiration dates of OTC medications in your medication cabinet?	Yes 162 (81.0)	Unsure 2 (1.0)	No 36 (18.0)
Have you ever shared your OTC medications with someone else?	Yes 132 (66.0)	Unsure 2 (1.0)	No 66 (33.0)

**Table 7:** Participants’ level of behaviour towards OTC medications.

Statement	Mean	SD	Level of Behavior
Do you keep track of the expiration dates of OTC medications in your medication cabinet?	2.63	0.772	Responsible behaviour
Have you ever shared your OTC medications with someone else?	1.67	0.941	Risky behaviour
Overall Mean Behavior Score	2.22		

**Table 8:** Association between socio-demographic factors and participants’ knowledge level regarding OTC medications.

Demographics	Level of Knowledge			*p*-Value
	Inadequate Knowledge n (%)	Moderately Adequate Knowledge n (%)	Adequate Knowledge n (%)	
Age (years)				
From 18 to 25 year	2 (33.3)	22 (19.0)	17 (21.8)	0.149
From 26 to 35 year	1 (16.7)	29 (25.0)	28 (35.9)	
From 36 to 45 year	3 (50)	36 (31.0)	18 (23.1)	
From 46 to 55 year	0 (0.0)	17 (14.7)	13 (16.7)	
Above 55 years	0 (0.0)	12 (10.3)	2 (2.6)	
Gender				
Male	5 (83.3)	84 (72.4)	51 (65.4)	0.435
Female	1 (16.7)	32 (27.6)	27 (34.6)	
Education Level				
High School or less	1 (16.7)	13 (11.2)	15 (19.2)	0.060
Diplomas’ degree	0 (0.0)	20 (17.2)	5 (6.4)	
Bachelor’s degree	5 (83.3)	59 (50.9)	45 (57.7)	
Higher education	0 (0.0)	24 (20.7)	13 (16.7)	

**Table 9:** Association between socio-demographic factors and participants’ attitude level regarding OTC medications.

Demographics	Level of Attitude		*p*-Value
	Neutral n (%)	Positive Attitude n (%)	
Age (years)			
From 18 to 25 year	28 (24.8)	13 (14.9)	0.131
From 26 to 35 year	34 (30.1)	24 (27.6)	
From 36 to 45 year	31 (27.4)	26 (29.9)	
From 46 to 55 year	16 (14.2)	14 (16.1)	
Above 55 years	4 (3.5)	10 (11.5)	
Gender			
Male	78 (69.0)	62 (71.3)	0.732
Female	35 (31.0)	25 (28.7)	
Education Level			
High School or less	20 (17.7)	9 (10.3)	0.427
Diplomas’ degree	14 (12.4)	11 (12.6)	
Bachelor’s degree	61 (54.0)	48 (55.2)	
Higher education	18 (15.9)	19 (21.8)	

## Data Availability

The data are available upon request from the corresponding author.
